# Lymphopenia-induced lymphoproliferation drives activation of naive T cells and expansion of regulatory populations

**DOI:** 10.1016/j.isci.2021.102164

**Published:** 2021-02-07

**Authors:** S. Eldershaw, K. Verma, W. Croft, T. Rai, F.A.M. Kinsella, C. Stephens, H. Chen, J. Nunnick, J. Zuo, R. Malladi, P. Moss

**Affiliations:** 1 Institute of Immunology and Immunotherapy, University of Birmingham, Birmingham, UK; 2 Centre for Computational Biology, University of Birmingham, Birmingham, UK; 3 Center for clinical Haematology, Queen Elizabeth Hospital, Birmingham, UK

**Keywords:** Immunology, Cell Biology

## Abstract

Chemotherapy pre-conditioning is an essential component of chimeric antigen receptor transduced cell therapy. Acute lymphopenia-induced proliferation (LIP) is known to be driven primarily by homeostatic cytokines, but little is known on the underlying mechanisms in humans. We undertook phenotypic and transcriptional analysis of T cells undergoing LIP two weeks post-myeloablative autograft stem cell transplantation. Strong IL-7 signaling was reflected in downregulated IL-7R expression on all T cells, including naive cells, along with parallel increased IL-2Rα expression. Notably, activated residual naive cells expressed Fas indicating recent TCR engagement. Moreover, proportion of Ki67 + FoxP3+ Tregs was almost doubled. Transcriptional analysis revealed increased fatty acid metabolism and interferon signaling responses. In contrast, TGF-β signaling was strongly suppressed. Thus, human LIP response is characterized by cytokine and TCR-driven proliferation which drives global T cell activation but also preferentially triggers regulatory cell expansion which may limit tumor-specific immunity. These features indicate potential therapeutic opportunities to manipulate immunotherapy regimens incorporating LIP conditioning protocols.

## Highlights

• Lymphopenia drives global T cell activation with selective FoxP3+ T cell proliferation
• naive T cells undergo TCR-mediated activation and effector populations expand
• IL-7R is downregulated on all T cell subsets whilst IL-2Rα expression increases
• Fatty acid metabolism & interferon-signaling increase; TGF-β responses suppressed

## Graphical abstract

**Figure:**
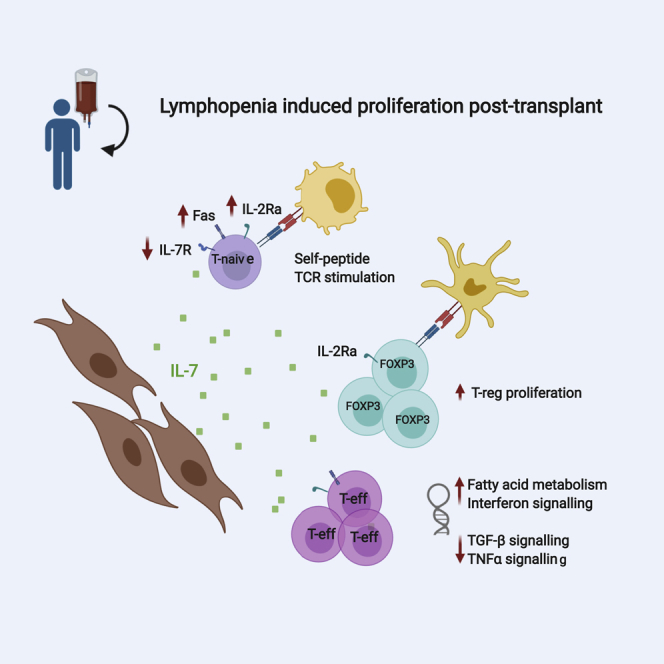

## Introduction

A characteristic feature of the immune system is the maintenance of a stable lymphoid pool with relatively little fluctuation in lymphocyte number over time. Homeostatic proliferation is one mechanism by which this is achieved and refers to the ability of lymphoid cells to increase proliferation during periods of acute and chronic lymphopenia (Takada and Jameson, 2009) (Mackall and Gress, 1997).

The homeostatic cytokines IL-7 and IL-15 are important mediators in this regard with IL-7 acting as a primary ‘rheostat’ through its proliferative and anti-apoptotic activity. IL-7 is produced by a range of stromal cells and serum concentrations are regulated through consumption by lymphoid populations (Martin et al., 2017). IL-7 concentrations therefore increase during lymphopenia (Condomines et al., 2010) and act to drive increased lymphoproliferation (Perales et al., 2012). In contrast, IL-15 mediates its effect primarily on memory CD8+ T cells and natural killer cells. Profound lymphopenia leads to lymphopenia-induced proliferation (LIP) and in this setting murine models indicate that T cell receptor engagement also promotes proliferation through recognition of self-peptides by naive T cells (Winstead et al., 2010) (Goldrath et al., 2000) (Cho et al., 2016).

Chemotherapy and conditioning regimens used prior to stem cell transplantation lead to profound lymphopenia and mediate a state of acute LIP characterized by intense T cell proliferation. However, the underlying mechanisms that drive this remain uncertain. T cell adoptive therapy, using approaches such as chimeric antigen receptor transduced (CAR-T) cells (Restifo et al., 2012) (Brentjens et al., 2013) (Gattinoni et al., 2005), has transformed the outlook for many patients with malignant disease but the success of this approach is reliant on generation of administration of chemotherapy in order to promote LIP.

Here we undertook a detailed assessment of the phenotypic and transcriptional profile of T-cells following hemopoietic autografting as a model to improve understanding of mechanisms that underlie acute LIP in the human setting. Our hypothesis was that this would be mediated by enhanced TCR and cytokine signaling although details of these remain uncertain and we anticipated that this data set would reveal novel biochemical and transcriptional mechanisms that underlie the proliferative response of T cells in this setting. The translational ambition was that this could be of value in the development of novel CAR-T regimens that facilitate LIP without the use of toxic conditioning regimens. We report that T cells display intense global proliferation, including naive cell activation, mediated by an interferon-driven proliferation fueled by fatty acid metabolism. Unexpectedly, this is associated with enhanced transcription of genes associated with regulatory function and an increase in the proportion of FoxP3+ T regulatory cells. These findings provide new insights into the mechanism of LIP in the human immune system and potentially offer a range of novel translational opportunities.

## Results

### T cells numbers are very low in first two weeks after autograft transplantation but cells are undergoing intense proliferation

Initial studies were undertaken to determine the T cell count in patients during the early period after hemopoietic autograft transplantation. Blood samples of individual patients were analyzed longitudinally, taken immediately prior to stem cell infusion (day 0) and then on day 7 and day 12 after transplant (n = 41, 58, and 56, respectively). The number of TCRαβ cells was determined using absolute lymphocyte count and flow cytometric analysis.

**Figure 1:**
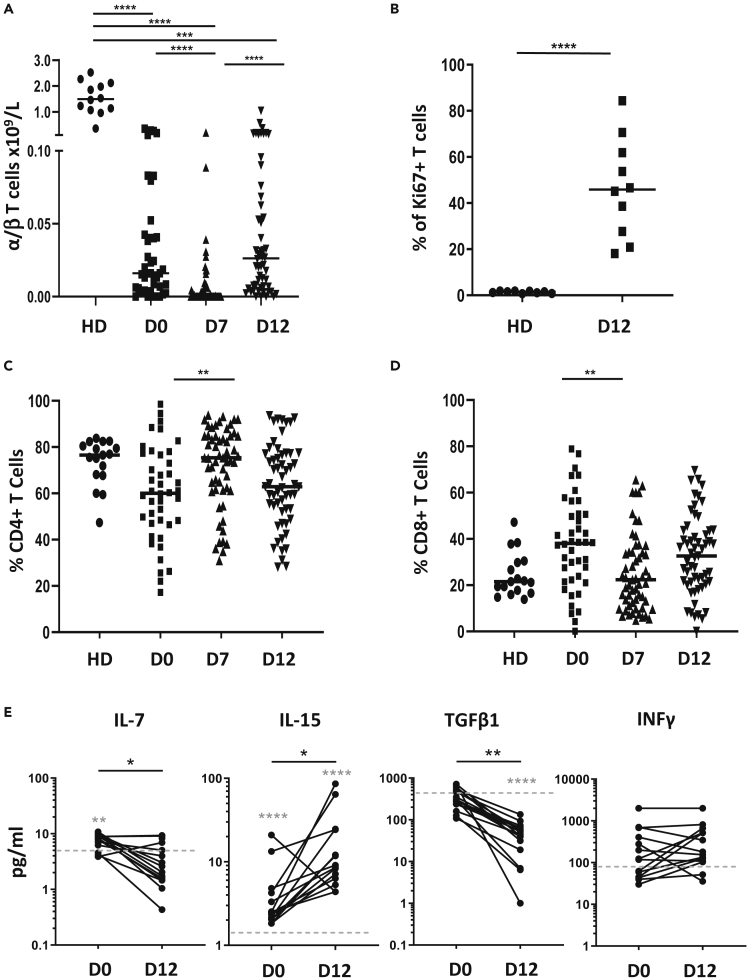
αβ T cell numbers are very low but undergoing intense proliferation in the early lymphopenic environment post-transplant (A) Absolute count of TCRab T cells at day 0 (D0, n = 41), at day 7 (D7, n = 58) and day 12 (D12, n = 56) post-transplant. Values from healthy donors are also shown (HD, n = 12). (B) Ki67 staining on T cells from HD (n = 9) and D12 patients (n = 10). (C and D) (C) Percentage of CD4+ and (D) CD8+ T cells within each group (HD, n = 17; D0, n = 42; D7, n = 57; D12, n = 57). (E) Serum concentration of cytokines IL-7, IL-15, TGFβ and IFNƴ measured in matched D0 and D12 patient samples (n = 14). Gray line depicts the median value in healthy controls. Kruskal-Wallis test with Dunn's multiple comparison test (A,C and D), Wilcoxon signed-rank test (E) and Mann-Whitney U test (B, E(gray)) was performed for statistical analysis, ∗∗p < 0.01, ∗∗∗p < 0.001 and ∗∗∗∗p < 0.0001.

T cell counts were low on day 0 (0.016 x 10^9^/l) compared to age-matched healthy donors (1.5 x 10^9^/l) and fell further during the first week to undetectable levels. Numbers then significantly increased over the next few days to reach a median value of 0.026 x 10^9^/l on day 12 (Figure 1A). Ki-67 staining was used to measure the proportion of T cells that were undergoing active division. This was measured at 46% of total T cells at day 12 compared to only 1.5% in healthy donors (Figure 1B). T cells are therefore undergoing a very high degree of LIP within the early post-transplant period.

We next determined the relative proportion of CD4+ and CD8+ T cells which represented 60% and 38% of T cells, respectively, on day 0. The proportion of CD4+ cells significantly increased to 75% by day 7 before falling back to 62% on day 12 (Figure 1C). A reciprocal pattern was observed for CD8+ populations (Figure 1D), suggesting that CD4+ T cells have a brief selective proliferative advantage in the first week post-transplant. Nevertheless, no overall difference in the CD4:CD8 ratio was observed in the first two weeks post-transplant.

ELISA was used to determine the serum concentration of IL-7, IL-15, TGF-β1, and IFN-γ during the first 12 days post-transplant (Figure 1E). IL-7 fell from 8.6pg/ml at the time of transplant to a median of 2.6pg/ml at day 12, compatible with peripheral consumption during LIP. In contrast IL-15 concentration at day 0 was 2.5pg/ml, above median values in HD (1.6pg/ml), and increased further to 8.8pg/ml at day 12. TGF-β1 serum levels fell markedly over the first 12 days post-transplant (301.3pg/ml to 45.1pg/ml) but no changes were observed in IFN-γ concentration.

### The proportion of naive T cells falls dramatically after transplant whilst effector cells are substantially increased

**Figure 2:**
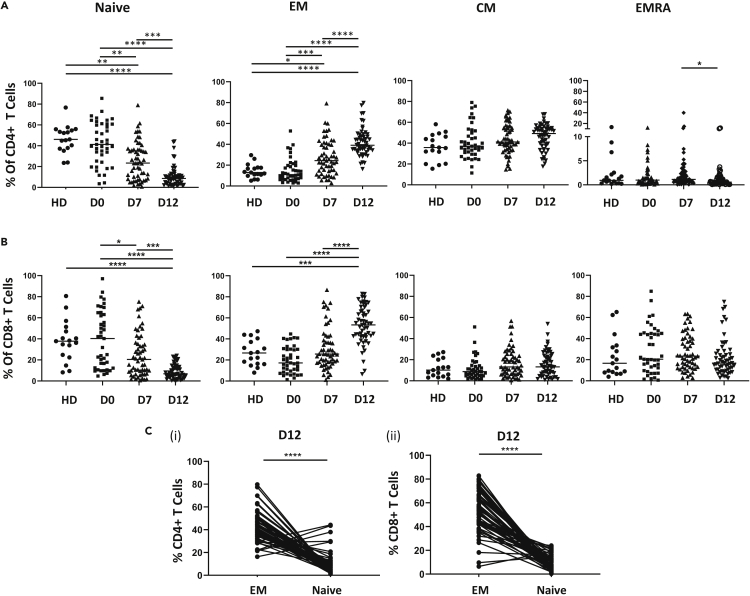
The proportion of naive T cells falls markedly after transplant whilst effector subsets increase (A and B)Percentage of T cell subsets in (A) CD4+ and (B) CD8+ population in healthy donors (HD, n = 17) and autograft patients at day 0 (D0 n = 41), at Day 7(D7, n = 57) and Day 12 (D12, n = 56) post-transplant. (C) Paired comparison of proportions of CD4+ (i) and CD8+ (ii) naive and effector memory (EM) subsets in each patient at day 12 post-transplant (n = 56). Bar indicates median value. Kruskal-Wallis test with Dunn's multiple comparison test (A,B) and Wilcoxon matched-pairs signed-ranks test (C) was performed for statistical analysis, ∗p < 0.05, ∗∗p < 0.01, ∗∗∗p < 0.001, ∗∗∗∗p < 0.0001.

The expression of CD45RA and CCR7 was then used to delineate the proportion of naive (CD45RA + CCR7+), central memory (CD45RA-CCR7+), effector (CD45RA-CCR7-) and effector memory (CD45RA + CCR7-) cells. The most striking observation was that the proportion of naive cells fell substantially within the first two weeks post-transplant. In particular, these represented 37% of CD4+ cells on day 0 but only 8.6% at day 12 (Figure 2A). The comparable values for CD8+ subsets were 37% and 6.9%, respectively (Figure 2B). This reduction was mirrored by a substantial increase in the proportion of effector cells (EMs) during this period, rising from 11% to 39%, and 18%–53%, within the CD4+ and CD8+ populations, respectively. No significant differences were seen in the relative proportion of central memory or EMRA cells during this period. In order to determine the direct relationship between the proportion of naive and effector memory, we also assessed the relative change within individual patients and a strong correlation was observed (Figure 2C).

### Fas expression on naive T cells reveals recent TCR engagement during LIP

**Figure 3:**
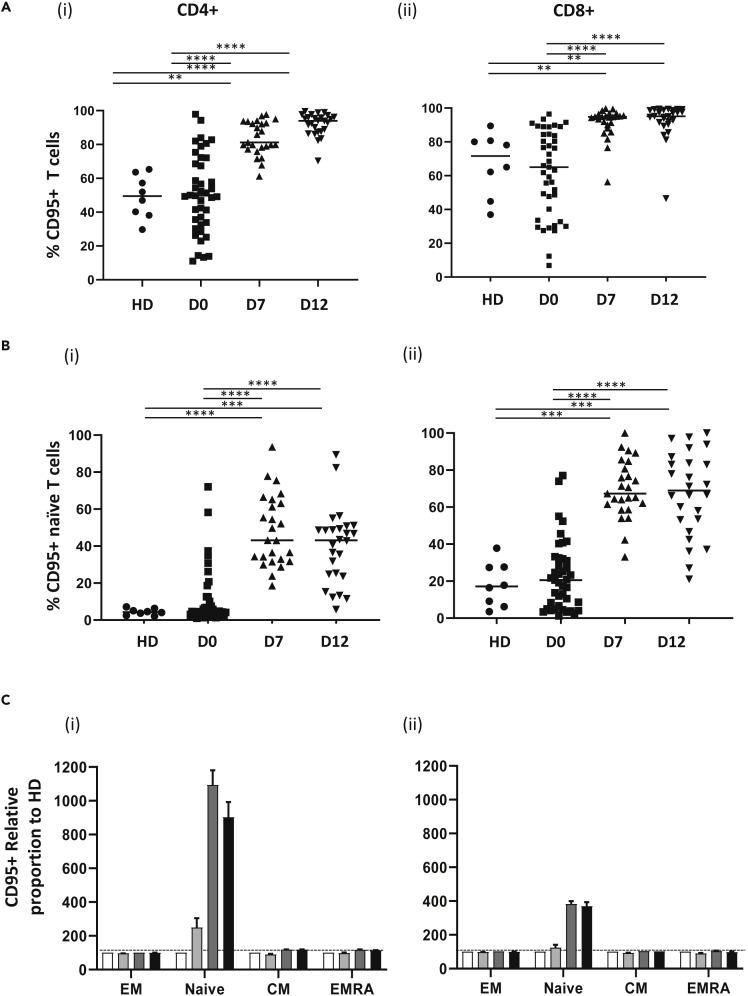
Fas expression is markedly upregulated on naive T cells during lymphopenia-induced proliferation (A) Fas (CD95) expression on CD4+ (i) and CD8+ (ii) T cells in healthy donors (HD, n = 8) and in patients at day 0 (D0, n = 41), Day 7 (D7, n = 25) and day 12 (D12, n = 26) post-transplant. (B) Proportion of naive CD4+ (i) and naive CD8+ (ii) T cells expressing CD95 in healthy donors (HD, n = 8) and in patients at D0 (n = 41), D7 (n = 25) and D12 (n = 26) post-transplant. (C) Mean proportion of CD4+ and CD8+ T cell subsets expressing CD95 in patient samples relative to healthy donors (where HD = 100% represented as dotted line) in CD4+ (i) and CD8+ (ii) T cells (HD; white bars, D0; light gray bars, WK1; dark gray bars, WK2; black bars). Error bars depict standard error mean (SEM). Kruskal-Wallis with Dunn's multiple comparison test was performed, ∗∗p < 0.01, ∗∗∗p < 0.001, ∗∗∗∗p < 0.0001.

Our previous findings, showing a marked reduction in the proportion of naive T cells, led us to investigate if cells with a naive phenotype had undergone TCR-mediated stimulation and differentiation during LIP. As such, we examined the expression profile of Fas (CD95) in relation to memory profile. Fifty percent and 64% of global CD4+ and CD8+ T cells expressed Fas at the time of transplant but this increased substantially to reach respective levels of 94% and 96% at D12 (Figure 3A). Assessment of Fas expression in relation to differentiation status revealed a 9.3-fold and 3.5-fold increase of Fas expression on naive CD4+ and CD8+ T cells, respectively. Indeed, Fas expression was seen on over 43% and 69% of phenotypically ‘naive’ CD4+ and CD8+ T cells, respectively, in the early post-transplant period (Figure 3B). In contrast, no increase in Fas expression was seen on effector or memory subsets in keeping with the stable expression of this marker on antigen-experienced cells (Figure 3C).

### T cells undergoing LIP display a reciprocal pattern of IL-7R and IL-2Rα expression

Given the importance of common gamma chain cytokines in homeostatic proliferation we next measured the profile of IL-7R (CD127) and IL-2Rα (CD25) expression on T cell subsets during LIP (Figure S1). IL-7R is downregulated following engagement with its ligand (Francois et al., 2018) and therefore a reduction in expression is representative of recent cytokine activation.

**Figure 4:**
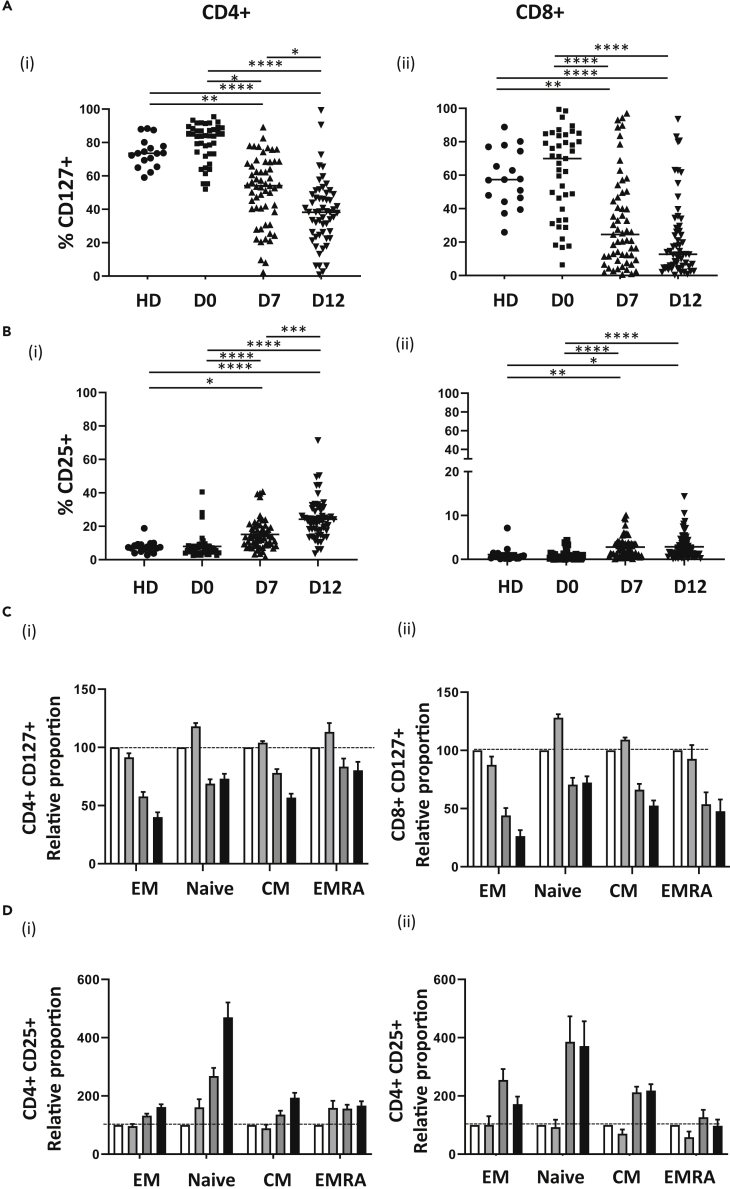
Reciprocal pattern of expression of IL-7R and IL-2Rα during lymphopenia-induced proliferation CD127 (A) and CD25 staining (B) on CD4+ (i) and CD8+ (ii) T cells from healthy donors (HD, n = 17) and autograft patients at day 0 (D0, n = 41) and day 7 (D7, n = 57) and day 12 (D12, n = 56) post-transplant. The mean proportion of CD4+ and CD8+ T cell subsets expressing CD127 and CD25 was calculated for healthy donors (n = 17) and patients at day 0 (n = 41), D7 (n = 57) and D12 (n = 56) post-transplant. The percentage expression for the patient samples at the three time points was then calculated relative to HD (where HD = 100%) for CD127 (C) and CD25 (D) on CD4+ (i) and CD8+ (ii) T cells (HD; white bars, D0; light gray bars, D7; dark gray bars, D12; black bars). Error bars depict standard error mean (SEM). Kruskal-Wallis with Dunn's multiple comparison test was performed, ∗p < 0.05, ∗∗p < 0.01, ∗∗∗p < 0.001, ∗∗∗∗p < 0.0001.

IL-7R expression fell markedly during the first two weeks post-autograft in both the CD4+ and CD8+ T cell pools. Indeed, the proportion of IL-7R-positive cells decreased by around 50% in both subsets, albeit from a slightly higher starting level of 82% in CD4+ populations compared to 68% in the CD8+ pool (Figure 4A). In contrast, IL-2Rα expression was seen to be increased in both subsets, particularly in the CD4+ pool. Within CD4+ cells, values rose 5-fold, from 3.6% on day 0–19% at day 12, whilst the percentage increase was from 0.1% to 1%, respectively, within the CD8+ population (Figure 4B).

We next examined the expression of IL-7R and IL-2Rα on naive, central memory, effector, and effector memory pools compared to healthy donors. IL-7R expression fell on all memory subsets during the first 2 weeks, and this was particularly marked on EMs (Figure 4C). In contrast, IL-2Rα expression increased during this period, most particularly within the naive pool where the proportion of CD25 + cells increased by 4.7 and 3.6-fold, respectively, in the CD4+ and CD8+ repertoires (Figure 4D).

### LIP favors expansion of regulatory CD4+ and CD8+ T cells

**Figure 5:**
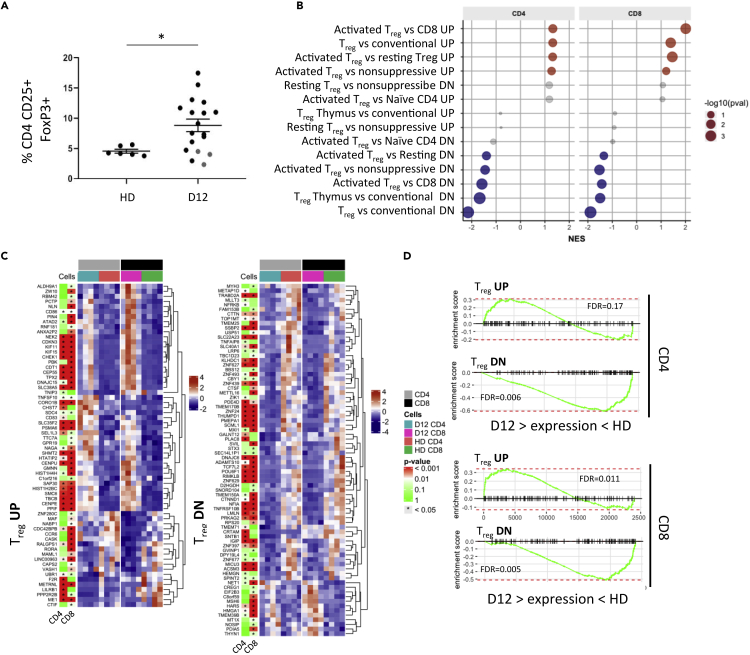
Lymphopenia-induced proliferation drives T cells toward a regulatory phenotype (A) Proportion of CD4+CD25 + CD127-FoxP3+ T cells in healthy donors (HD n = 6) and autograft patients at day 12 post-transplant (n = 18) (∗p = 0.023). (B) Gene set enrichment analysis (GSEA) of relevant published T_reg_ signature gene sets. RNA sequencing was performed on FACS-sorted CD45RA-CCR7- CD4 and CD8 effector T cells of healthy donor (HD; n = 4) and day 12 post-autograft patients (D12; n = 4). Differential expression analysis performed for D12 vs HD on CD4 and CD8 effector T cell subset data separately. GSEA results show normalized enrichment score (NES) for selected gene sets and colored circles indicate significant enrichments toward genes upregulated (Red) or downregulated (blue) in CD4/CD8 effector T cells at D12 compared to HD. (C) Gene expression profiles of published signature genes (GSE22045 – genes UP/DN-regulated in T_reg_ cells compared to conventional T cells) observed in FACS-sorted effector T cells at D12 compared to HD. Shown are genes from this gene set that are differentially expressed genes between D12 and HD in at least one cell type (CD4/CD8). p values are from differential expression analysis of D12 vs HD CD4/CD8 and are adjusted for multiple testing. Color key = standard deviation from the mean normalized expression level. (D) Barcode plots from gene set enrichment analysis of gene sets in C showing enrichments on the fold change expression ranking from D12 vs HD differential expression analysis.

Downregulation of IL-7R (CD127) and high levels of IL-2Rα (CD25) are characteristic features of regulatory T cells (Liu et al., 2006). As such we were next interested to assess if T cells were driven toward a regulatory phenotype during LIP. Tregs were identified by the phenotype CD25 + CD127-FOXP3+ within the CD4+ and CD8+ T cell population. The percentage of CD4+ Tregs was 8.3% at day 12, nearly double the value of 4.2% in HD (Figure 5A). FoxP3+ CD8+ T cells were not detected (data not shown). FOXP3+ CD4+ T cells were more proliferative than FOXP3- CD4+ T cells at D12 post-transplant as shown by Ki67 + staining (Figure S2). There was an increase in FOXP3+ CD4+ T cells at D12 compared to D0 (Figure S3), but there was no significant difference between percentage apoptotic or percentage live CD25 + CD4+ T cells between D0 and D12 post-transplant (Figure S2). In addition, we also interrogated the transcriptional profile of T EM populations at day 12 compared to EMs from healthy donors. Gene set enrichment analysis (GSEA) showed strong correlations between gene expression in EMs at day 12 and a range of data sets from activated Tregs subsets (Figure 5B). Indeed, a wide range of individual genes that are differentially expressed in Tregs were seen to exhibit a similar profile in day 12 EMs (Figure 5C). Barcode plots from gene set enrichment analysis further confirmed the correlation of day 12 transcriptional signature with that reported in T regulatory subsets, particularly in relation to the profile of downregulated genes (Figure 5D).

### Transcriptional profiling identifies signaling pathways that drive LIP

**Figure 6:**
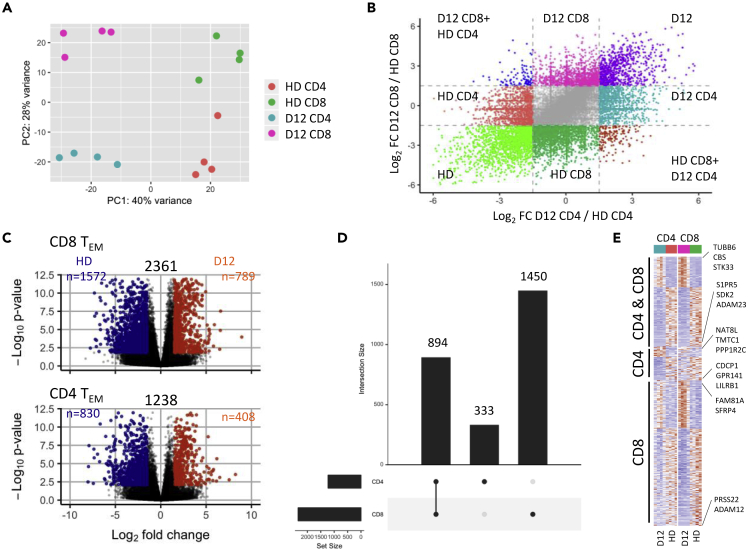
Transcriptional profiling of effector T cells during lymphopenia-induced proliferation (A) Transcriptomes of FACS-sorted CD45RA-CCR7- CD4 and CD8 effector T cells of healthy donor and day 12 post-autograft patients in reduced dimensional space. Principal component coordinates generated by principal component analysis on normalized gene count data from RNA sequencing. (B) Overview of gene-level transcriptional changes from calculated fold changes in gene expression at day 12 compared to HD for CD4 and CD8 effector T cells. (C) Differential expression analysis results from D12 vs HD comparisons of CD4 and CD8 cells. Colored points indicate differentially expressed genes in the comparison (Orange = UP at D12; Blue = DOWN at D12). Genes are deemed to differentially expressed if adjusted p value <0.05 and absolute log2FC > 1.5. (D and E) (D) Overlaps of differentially expressed genes between CD4 and CD8 cells (E) Gene expression profiles of differentially expressed genes from C. Color indicates standard deviation from mean (red = increase at D12; blue = decrease at D12). Genes are grouped into those identified as differentially expressed between D12 and HD samples in both CD4 and CD8, CD4-only or CD8-only effector T cells. The top three UP/DN differentially expressed genes in each group (ordered by fold change) are labeled.

In order to investigate the biochemical basis for LIP, we further interrogated transcriptional analysis of FACS-sorted CD4+ and CD8+ T EMs (CD45RA-CCR7-) from patients at day 12 compared to those from healthy donors. Principal component analysis (PCA) and hierarchical clustering analysis revealed discrete clustering of CD4+ and CD8+ populations from patients or healthy donors with PC1 axis separating D12 from HD and PC2 axis separating CD4 from CD8 subsets (Figures 6A and S4). Greater transcriptional differences were present between CD4+ and CD8+ T cells at D12 compared to HD indicating some differential mechanisms of acute LIP between these two subsets (Figure S4). Indeed, nearly twice as many genes exhibited LIP-driven differential expression within CD8+ subsets compared to CD4+ cells (2361 and 1238, respectively) and downregulation of gene expression was around two-fold more common than upregulation in both subsets (Figures 6B and 6C). Eight hundred ninety four genes were differentially regulated in both the CD4+ and CD8+ signatures, whereas expression of 333 and 1450 genes was uniquely modulated in either CD4+ or CD8+ cells, respectively (Figure 6D). Alignment of individual genes allowed detection of the most strongly upregulated or downregulated genes in the ‘CD4+CD8’, ‘CD4-only’, and ‘CD8-only’ transcriptional profiles in order to facilitate study of shared and differential responses to LIP in CD4 and CD8 subsets. Genes most strongly upregulated in both subsets included Tubulin 6 (TUBB6), which is known to play a role in reorganization of the cytoskeleton during division and migration of immune cells (Sumoza-Toledo and Santos-Argumedo, 2004); CBS, a molecule involved in the biosynthesis of hydrogen sulfide which is a strong enhancer of T cell activation (Miller et al., 2012) and the serine threonine kinase STK33. Strongly downregulated genes in both subsets included the metalloprotease domain containing ADAM23, adhesion molecule SDK2, and sphingosine 1-phosphate receptor S1PR5. Modulation of Sphingosine 1-phosphate signaling is of particular note given its important role in directing T cell migration between lymphoid organs and circulatory fluids (Baeyens et al., 2015). Subset-specific gene expression changes of particular interest include downregulation of the CD6 ligand CDCP1 and the innate immune receptor for self LILRB1 on CD4 cells. Wnt/β-catenin signaling is required for the suppression of terminal differentiation and is essential for T cell memory formation (Gattinoni et al., 2010) and interestingly we observe strong increased expression in the Wnt/β-catenin signaling regulator molecule SFRP4 in the CD8-only subset.

**Figure 7:**
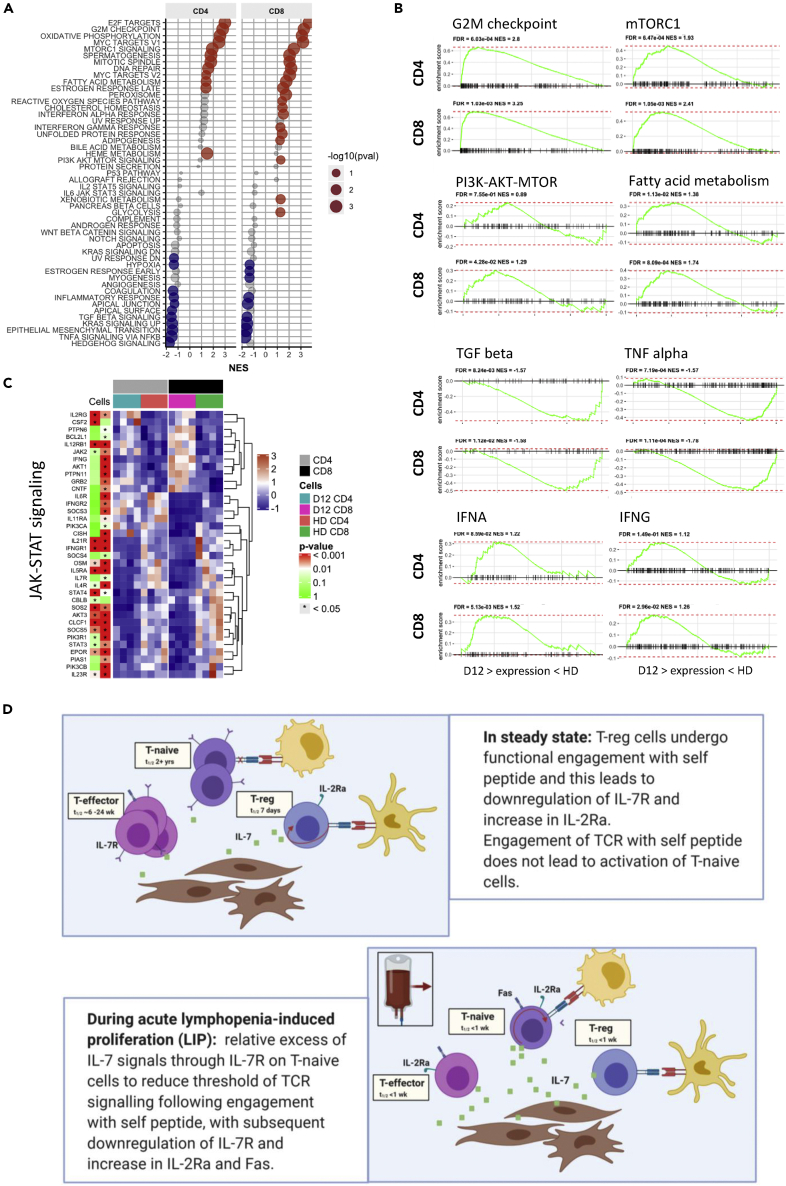
Effector T cells during lymphopenia-induced proliferation are undergoing fatty acid metabolism, show signatures of increased IFNA, IFNG, mTORC, and PI3K response and reduced TGFbeta and TNFalpha response compared to healthy donor (A) GSEA of selected MSigDB Hallmark gene sets. Shown are selected sets having FDR <0.2 in either CD4 or CD8 D12 vs HD comparisons. Colored circles indicate a significant enrichments (FDR<0.1) toward genes upregulated (Red) or downregulated (blue) at D12 compared to HD. (B) Selected significantly enriched gene sets from A including genes upregulated in G2M transition (cell-cycle progression) and fatty acid metabolism and in response to stimulation of the signaling pathways IFNA, IFNG, TGF beta, and TNF alpha. FDR = false discovery rate; NES = normalized enrichment score. (C) Gene expression profiles of UP/DN-regulated JAK-STAT signaling pathway genes in effector T cells at D12 post-autograft (D12) compared to healthy donors (HD). Showing D12 vs HD differentially expressed genes (in at least one cell type). p values are from differential expression analysis of D12 vs HD samples for each cell type and are adjusted for multiple testing. Color key = standard deviation from the mean normalized expression level. (D) Schematic diagram representing mechanisms of cytokine-driven T cell proliferation during LIP. Created using Biorender.

GSEA of selected MSigDB hallmark gene sets identified a wide range of upregulated pathways including oxidative phosphorylation and transcriptional targets of *E2F* and *MYC* (Figures 7A and S5). A strong proliferative profile was seen in LIP cells with activation of the nutrient sensor mTORC1 pathway and PI3/Akt signaling. Of note, genes associated with fatty acid metabolism were strikingly increased although the glycolysis pathway was increased in CD8+ cells only. Pathways associated with interferon-α and interferon-γ signaling were both activated whilst genes associated with TNF-α and TGF-β signaling were strongly downregulated during LIP (Figure 7B). Analysis of the JAK/STAT signaling pathway revealed differential expression of many genes, including upregulation of *JAK2* but reduced expression of *STAT3* and *STAT4* during LIP (Figure 7C). Transcription factor pathway analysis showed that genes regulated by NRSF were upregulated during LIP. Finally, many miRNA-target gene sets were downregulated (Figure S6) and overall a substantial proportion of non-coding RNA molecules were differentially expressed during LIP (Figure S7).

## Discussion

Homeostatic processes maintain the peripheral lymphocyte count at a remarkably stable level across the lifecourse. LIP acts to sustain lymphoid numbers and as chemotherapy-induced LIP is an essential component of almost all current CAR-T regimens we were interested to examine the mechanisms that regulate this process.

T cell proliferation was seen to increase very early after chemotherapy and the T cell count increased to around 26,000/mL at day 12 post-transplant driven by extensive cell division and Ki-67 expression on 50% of cells. CD4+ and CD8+ T cell populations expanded at equal rates over the first two weeks post-transplant (Tan et al., 2002).

A striking feature was the collapse in the percentage of naive cells in the early post-transplant period as these were replaced by an effector T cell pool, and no signature of CD31 + recent thymic emigrants was seen in this early period (Figure S8). This finding supports observations in murine systems that LIP drives naive cells to undergo activation, including acquisition of short-term functional capacity (Murali-Krishna and Ahmed, 2000). The survival of naive T cells requires their continuous contact with self-peptide:MHC ligands (Murali-Krishna et al., 1999) and LIP appears to further drive this process into T cell activation. Indeed the enrichment of auto-reactive cells and clinical development of auto-immune disease is well recognized during homeostatic proliferation (Jones et al., 2009) (King et al., 2004).

IL-7 is the major rheostat of LIP (Bourgeois and Stockinger, 2006) (Guimond et al., 2009) and IL-7R (CD127) expression fell on all T cell memory subsets during LIP, including the naive pool. However, IL-7 signaling may not be the only mechanism leading to CD127 downregulation as this can also be observed following TCR signaling (Carrette and Surh, 2012). Interestingly, this decrease was mirrored by a comparable, albeit smaller, increase in IL-2Rα (CD25) expression and likely reflects reciprocal regulation of these receptors. This was most clearly observed within the naive T cell pool where a 4-fold increase in CD25 expression was observed compared to baseline. Of note, IL-7 directly increases expression of the IL-2Rα (Simonetta et al., 2014) and is the likely mechanism to explain this observation.

This IL-7R^low^ IL-2Rα^high^ phenotype is characteristic of regulatory T cells and we were therefore interested to determine the profile of regulatory T cells during LIP. Importantly, the proportion of FoxP3+CD4+ regulatory cells was seen to double to reach over 8% of the CD4+ T cell pool. Similar findings have been observed in the months following alemtuzumab therapy (Bloom et al., 2008) and in murine models (Bosco et al., 2006). Tregs have a high endogenous proliferation rate and expand preferentially after adoptive transfer (Fisson et al., 2003) (Tang et al., 2008). As such, this phenotypic and transcriptional data indicates that while there does not seem to be a survival advantage, there is selective global expansion of regulatory T cells post-transplant during LIP. TGF-β levels were markedly suppressed at day 12 after transplant and transcription activity associated with TGF-β signaling was strongly downregulated. As such, it appears unlikely that this reflects TGF-β-mediated peripheral conversion of EMs. Rather we suggest that homeostatic cytokines can themselves initiate partial regulatory conversion during LIP. Depletion of Treg populations can augment tumor-specific immune responses in murine models (Baba et al., 2012) (Saida et al., 2015) and as such our findings suggest that a similar approach may potentially act to further boost tumor-specific immune responses after autograft (North, 1982) or CAR-T infusion. Further, these data may guide improvement of strategies for altered expression of these cytokines and/or their cognate receptors in CAR T cells to enhance the persistence and thereby antitumor activity of these engineered cells (Yao et al., 2012) (Andersen et al., 2016). Clearly, this approach must be titrated carefully in order to limit potential auto-immune reactions (King et al., 2004) and Treg may also play a role in supporting clonal diversity of EMs during LIP (Winstead et al., 2010) (Bolton et al., 2015)

One of the most interesting observations was remarkable increase in Fas (CD95) expression on T cells during LIP. Fas is a marker of T cell engagement and was, as expected, expressed exclusively on effector/memory pools prior to transplant in keeping with its role as a major regulator of lymphoid survival. However, Fas became expressed on the majority of ‘phenotypically naïve’ populations and reveals that LIP induces human naive cells to undergo partial T cell activation, presumably through recognition of self-peptides presented by HLA (Hsieh et al., 2004) (Johnson and Jameson, 2010). It is interesting to speculate why naive T cells undergo activation on self-peptides during LIP. As discussed above, naive cells utilize engagement with self-peptide:MHC for survival but although TCR signaling is attenuated by high phosphatase expression (Cho et al., 2016) they remain highly sensitive to cytokine-mediated activation and it thus seems that the supra-physiological levels of IL-7 during LIP serve to trigger partial T cell activation.

Chemotherapy-induced LIP is clearly not a natural event but observations from extreme phenotypes can provide insights into immune regulation and our findings may have implications for regulation of the physiological regulatory T cell pool which is characterized by high levels of proliferation (Vukmanovic-Stejic et al., 2006), IL-7R^low^IL-2Rα^high^ phenotype and recognition of self-peptide (Cozzo et al., 2003). It is now clear that Tregs are strongly dependent on peripheral IL-7 engagement (Bayer et al., 2008) (Simonetta et al., 2012) (Kim et al., 2012) (Bayer et al., 2008), and these data therefore suggest that many of the features of physiological Treg cells are themselves driven by enhanced sensitivity to homeostatic cytokines.

Differential expression of a wide variety of genes was observed during LIP, and it was noteworthy that many of these defined selective processes within either CD4+ or CD8+ lineages. However, 894 transcripts underwent similar regulation within both subsets and may represent targets for future T cell engineering in order to facilitate ‘chemotherapy-free’ T cell expansion without inducing LIP. Genes associated with fatty acid metabolism were highly upregulated and IL-7 induces T cell expression of glycerol channel aquaporin 9 (AQP9) and promotes glycerol import for fatty acid esterification and triglyceride (TAG) synthesis (Cui et al., 2015) (Barra et al., 2010). However AQP9 expression was not increased during LIP although expression of genes such as *AGPS, GNPAT* and *TPI1* that encode enzymes that catalyze reactions converting DHAP for lipid biosynthesis was strongly increased.

Genes associated with IFN-α signaling were also strongly upregulated, with a more modest increase in IFN-γ transcriptional activity. mTORC1 transcription, a major rheostat of cellular energy signaling, was also strongly increased. An unexpected finding was strong transcriptional downregulation of the TGF-β pathway. TGF-β is a major negative regulator of IL-7 driven T cell proliferation (Nguyen and Sieg, 2017), but serum levels are low in the early post-transplant period (Kyrcz-Krzemien et al., 2011). TGF-β RII expression was downregulated in both CD4 and CD8 T cells, whilst expression of KLF10, a key transcriptional regulator of TGF-β RII(Papadakis et al., 2015) is increased. Inhibition of TGF-β signaling through expression of a dominant negative TGF-β RII strongly boosts the T cell memory pool (Lucas et al., 2000) and is being used as a mechanism to facilitate adoptive T cell expansion without the need for pre-conditioning therapy (Bollard et al., 2018). Several transcription factors were differentially regulated during LIP, including c-MYB, which acts to maintain the stem cell memory pool (Gautam et al., 2019), and the expression of genes upregulated by NRSF was also increased by day 12.

This comprehensive characterization of acute LIP within the human lymphoid system reveals remarkable levels of proliferation driven by homeostatic cytokines and TCR-mediated activation of naive cells. Furthermore, a shift toward a ‘regulatory’ IL-7R^low^IL-25Ra^high^ phenotype and transcriptional profile is observed across the whole repertoire and supports growing interest in IL-7 as a major regulator of Treg phenotype in health and disease. This increase in regulatory phenotype may underpin the utility of autograft transplantation in the management of auto-immune conditions but also suggests opportunities for therapeutic depletion in the setting of malignancy disease. These observations may also facilitate novel therapeutic opportunities to simplify conditioning regimens for patients undergoing adoptive T cell therapy.

### Limitations of the study

Chemotherapy-induced lymphopenia is not a natural phenomenon in humans but provides a setting to study the proliferative immune response to lymphopenia *in vivo*. A caveat of our study is that gene expression patterns were studied at the cell population level by bulk RNA sequencing, and it will be of interest in future studies to assess immune cells at the single cell level to further understand cellular heterogeneity and gene expression dynamics induced by the lymphopenic environment.

### Resource availability

#### Lead contact

Further information and requests for resources and data should be directed to the lead contact, Paul Moss, Institute of Immunology and Immunotherapy, University of Birmingham, Birmingham, UK (P.Moss@bham.ac.uk).

#### Materials availability

This study did not generate new unique reagents.

#### Data and code availability

The RNA sequencing data in this paper have been deposited in GEO:GSE150834 (https://www.ncbi.nlm.nih.gov/geo/query/acc.cgi?acc=GSE150834).

## Methods

All methods can be found in the accompanying Transparent Methods supplemental file.

## Supplemental information

Document S1. Transparent methods, figures S1–S8, and table S1
