# Lymphopenia-induced lymphoproliferation drives activation of naive T cells and expansion of regulatory populations

**DOI:** 10.1016/j.isci.2025.111958

**Published:** 2025-02-07

**Authors:** S. Eldershaw, K. Verma, W. Croft, T. Rai, F.A.M. Kinsella, C. Stephens, H. Chen, J. Nunnick, J. Zuo, R. Malladi, P. Moss

## Main text

(iScience *24*, 102164; March 19, 2021)

Due to an error during production, the author names in the originally published paper appeared inverted; i.e., the authors’ surnames were set as first names and the authors’ first initials were set as surnames. This has now been corrected online.

