# Supplementary material for: Lymphopenia-induced lymphoproliferation drives activation of naive T cells and expansion of regulatory populations
Source: iScience. 2021 Feb 7;24(3):102164. doi: 10.1016/j.isci.2021.102164 (PMC7907823; doi:10.1016/j.isci.2021.102164)
Supplement: Document S1. Transparent methods, figures S1–S8, and table S1 [file mmc1.pdf]

iScience, Volume *24*

Supplemental information

Lymphopenia-induced lymphoproliferation drives activation of naive T cells and expansion of regulatory populations

Eldershaw S, Verma K, Croft W, Rai T, Kinsella FAM, Stephens C, Chen H, Nunnick J, Zuo J, Malladi R, and Moss P

Supplemental Information

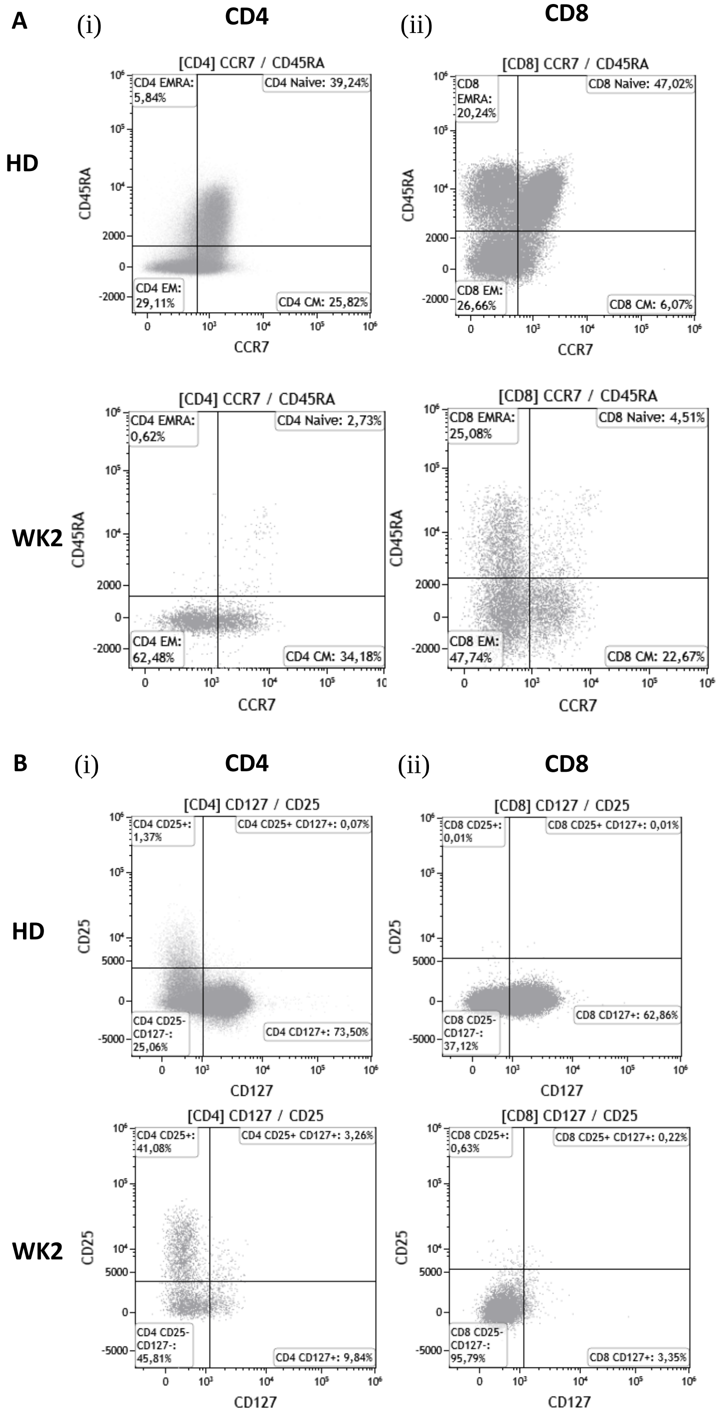

Figure S1. Autograft patients have a very different T cell phenotype at two weeks post-transplant compared to healthy donors, **Related to Figure 2** Representative flow plots of CD45RA and CCR7 staining for CD4 T cells and CD8 T cells from a healthy donor and patient at WK2 post-autograft. **(B)** Representative flow plots showing CD25 and CD127 staining.

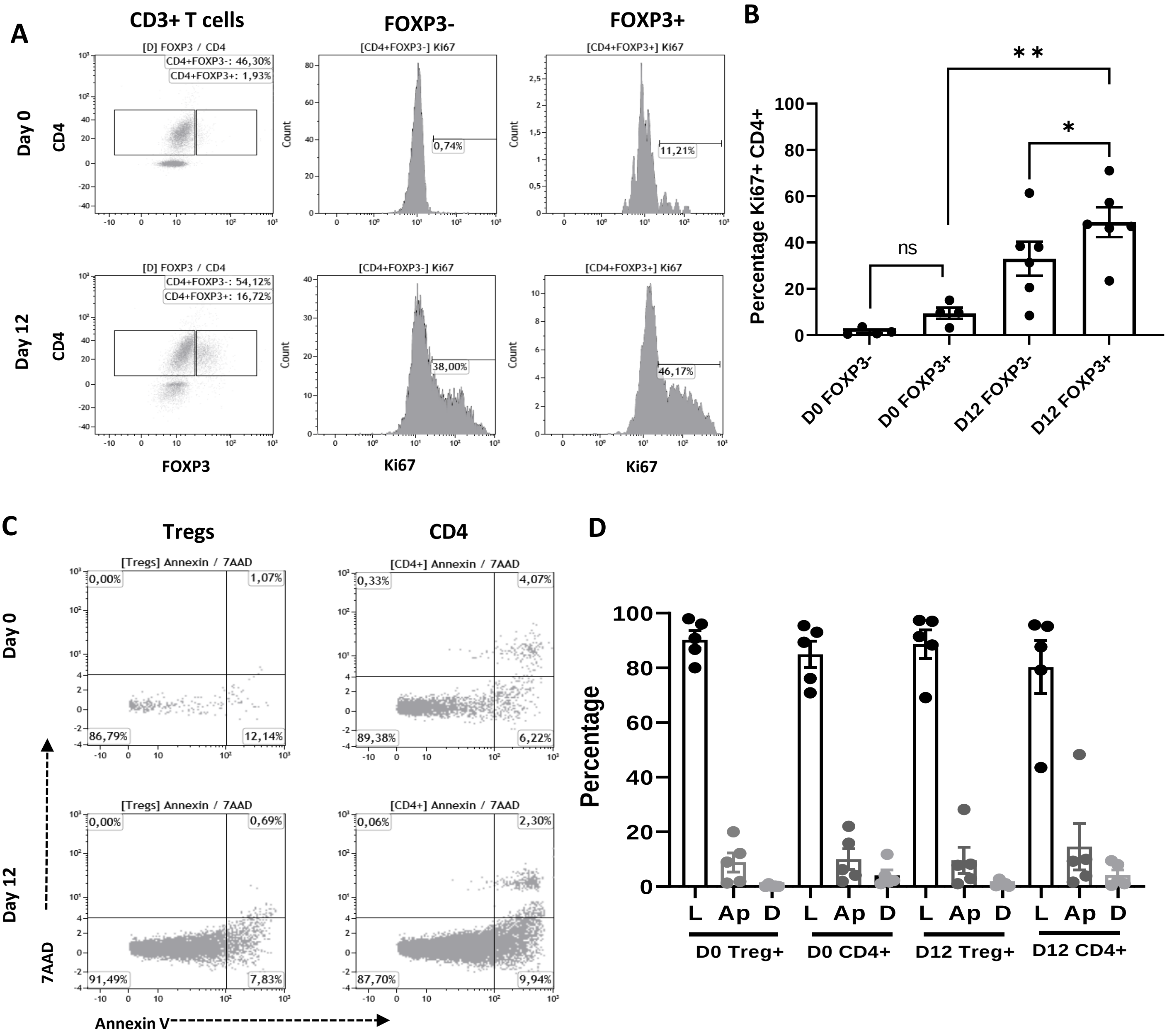

Figure S2. Increased proliferation of Tregs early after autologous stem cell transplant, Related to figure 5 (A) Representative FACS plot of paired D0 and D12 PBMCS showing FOXP3 staining on CD4 T cells and Ki67 staining on FOXP3- and FOXP3+ CD4 T cells. (B) Percentage of proliferating Ki67+ cells within the CD4+ FOXP3+ and CD4+FOXP3- T cells from blood samples collected at day 0 (D0, n=4) and day 12 (D12;. n=6) post-transplant. Statistical analysis was performed using Wilcoxon matched-pair signed rank test to compare FOXP3- and FOXP3+ for each time point. Mann-Whitney U test was performed to compare D0 and D12. (C) Representative FACS plots depicting Annexin V and 7AAD staining on paired D0 and D12 sample. (D)Percentage of Live(L; Annexin-7AAD-), Apoptotic (Ap; Annexin+7AAD-) and Dead (D; Annexin+7AAD+) cells within CD25+CD127- CD4+ T cells (Tregs) and whole CD4+ T cell population. No statistically significant differences were observed between the two time points. * represents p value <0.05, ** represents p value <0.01

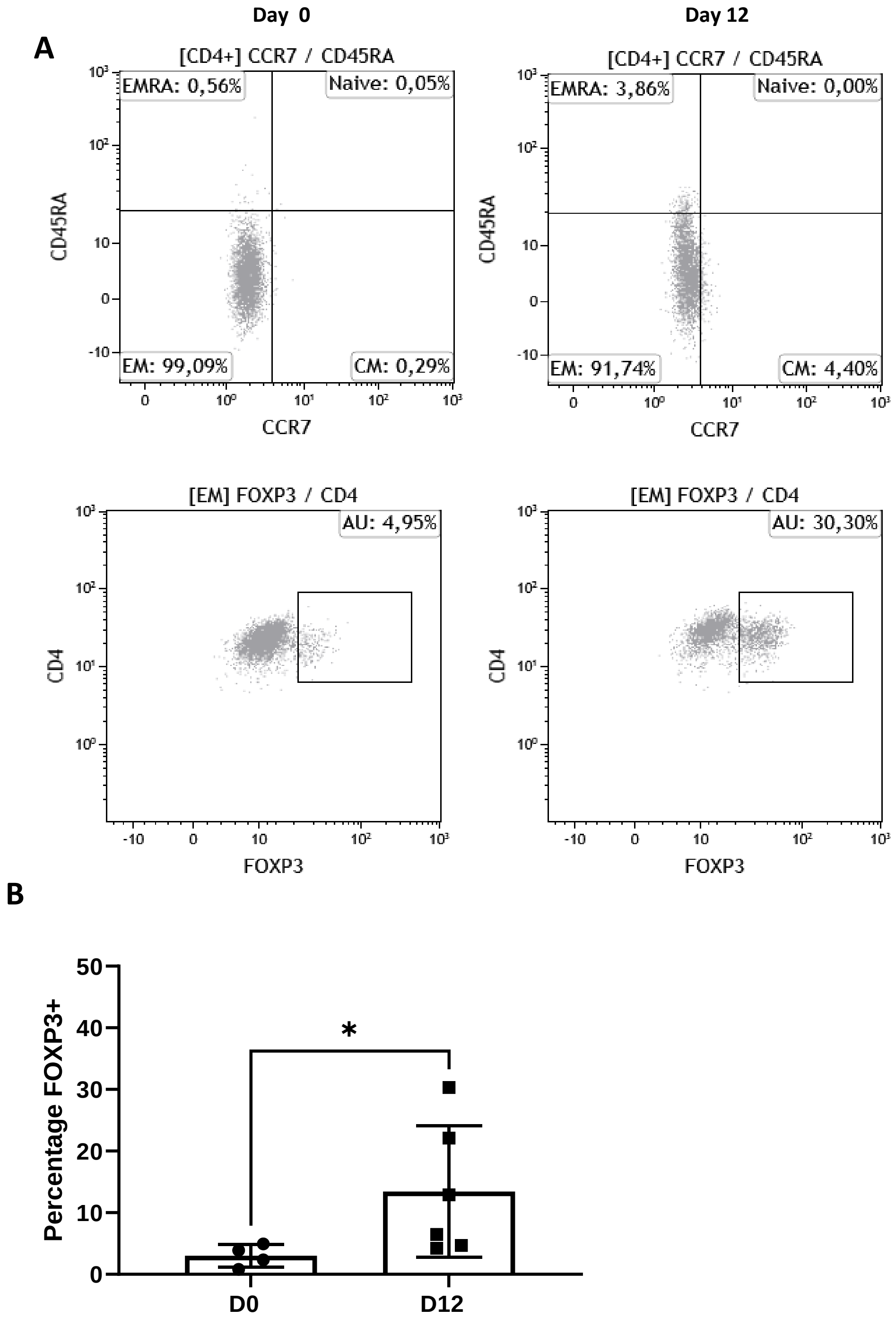

Figure S3. Proportion of FOXP3+ cells within EM CD4+ T cell population at D0 and D12 post-transplant, Related to figure 5 (A) Representative FACS plot of paired D0 and D12 PBMCS showing CD4 T cell subsets based on cell surface expression of CD45RA and CCR7 (upper panel) and FOXP3 staining (lower panel) on EM CD4+ T cells. (B) Percentage FOXP3+ cells within EM CD4+ T cells. Mann-Whitney U test was performed for statistical analysis. * represents p value <0.05

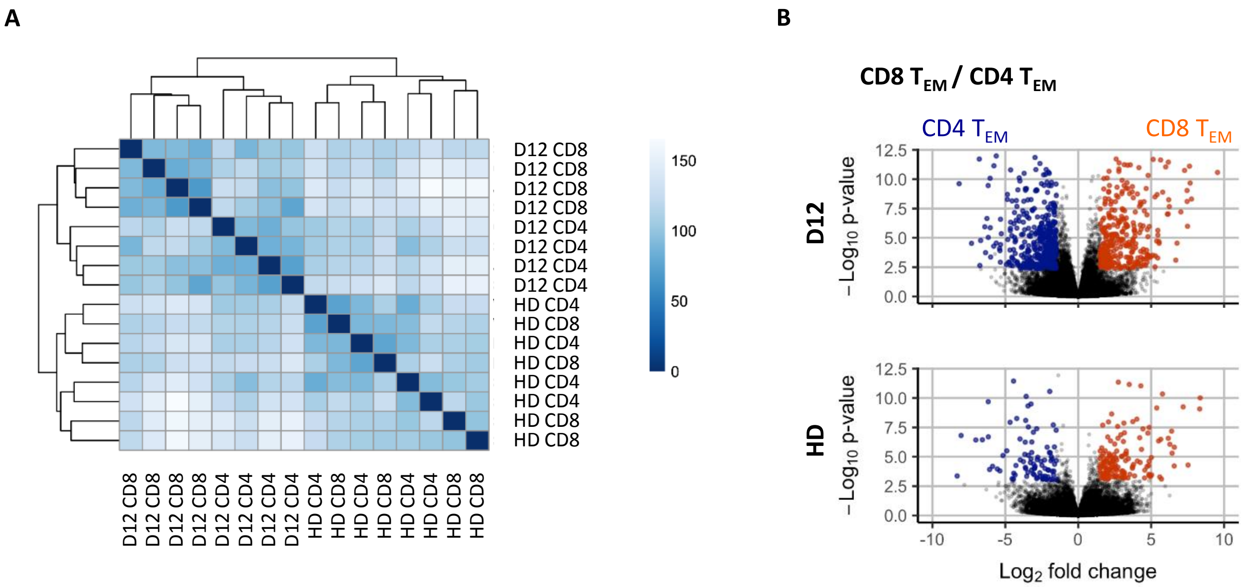

Figure S4. Sample distance matrix and Diversion of CD4 and CD8 T cell transcriptome at HD and at day 12 post autograft, Related to figure 5 (A) Sample distance matrix (Euclidean distance) of normalized gene expression data from RNA-seq of healthy donor (HD) and day 12 (D12) post autograft CD4 and CD8 effector T cells **(B)** Differential expression analysis results from CD8 vs CD4 effector T cell comparisons at day 12 or from healthy donors. Coloured points indicate differentially expressed genes in the comparison (Orange = UP in CD8 ; Blue=DOWN in CD8). Genes are deemed to differentially expressed if adjusted p-value <0.05 and absolute log2FC > 1.5.

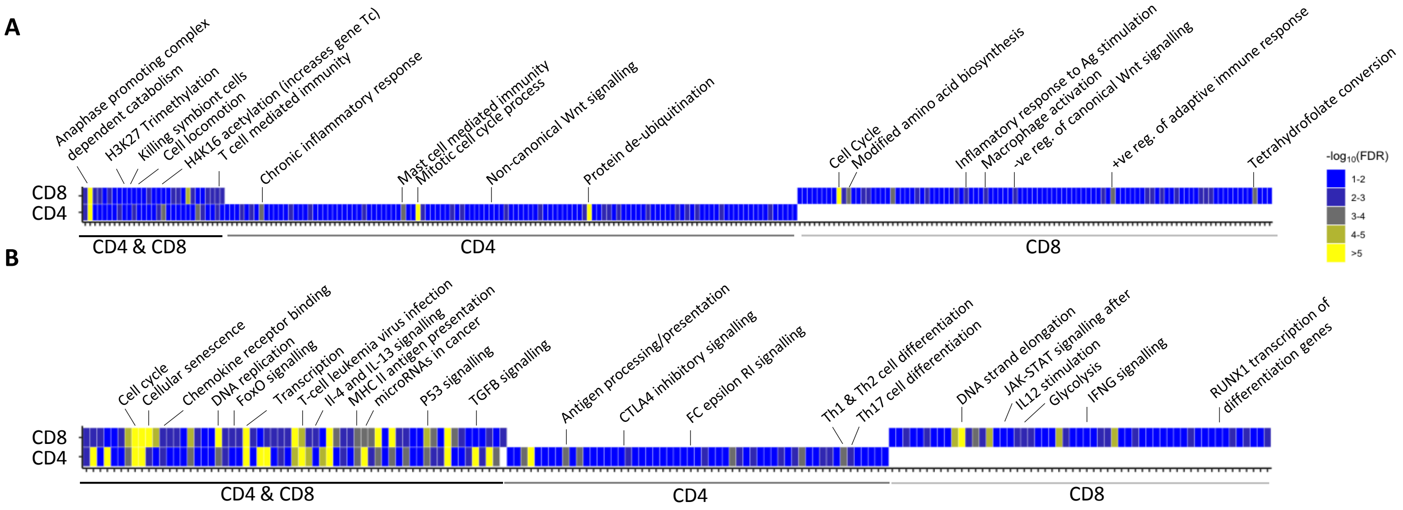

Figure S5. Functional enrichments within genes identified as being differentially expressed in effector memory T cells from patients at day 12 post-autograft vs healthy donors, Related to figure 6 Significant enrichments (FDR<0.1) of Biological Process **(A)** and Pathway **(B)** terms associated with genes differentially expressed in D12 CD4 or CD8 T cells compared to HD. Terms are ordered on the x axis from enrichments shared in both CD4 and CD8 DEGs to CD4 specific and CD8-specific DEGs

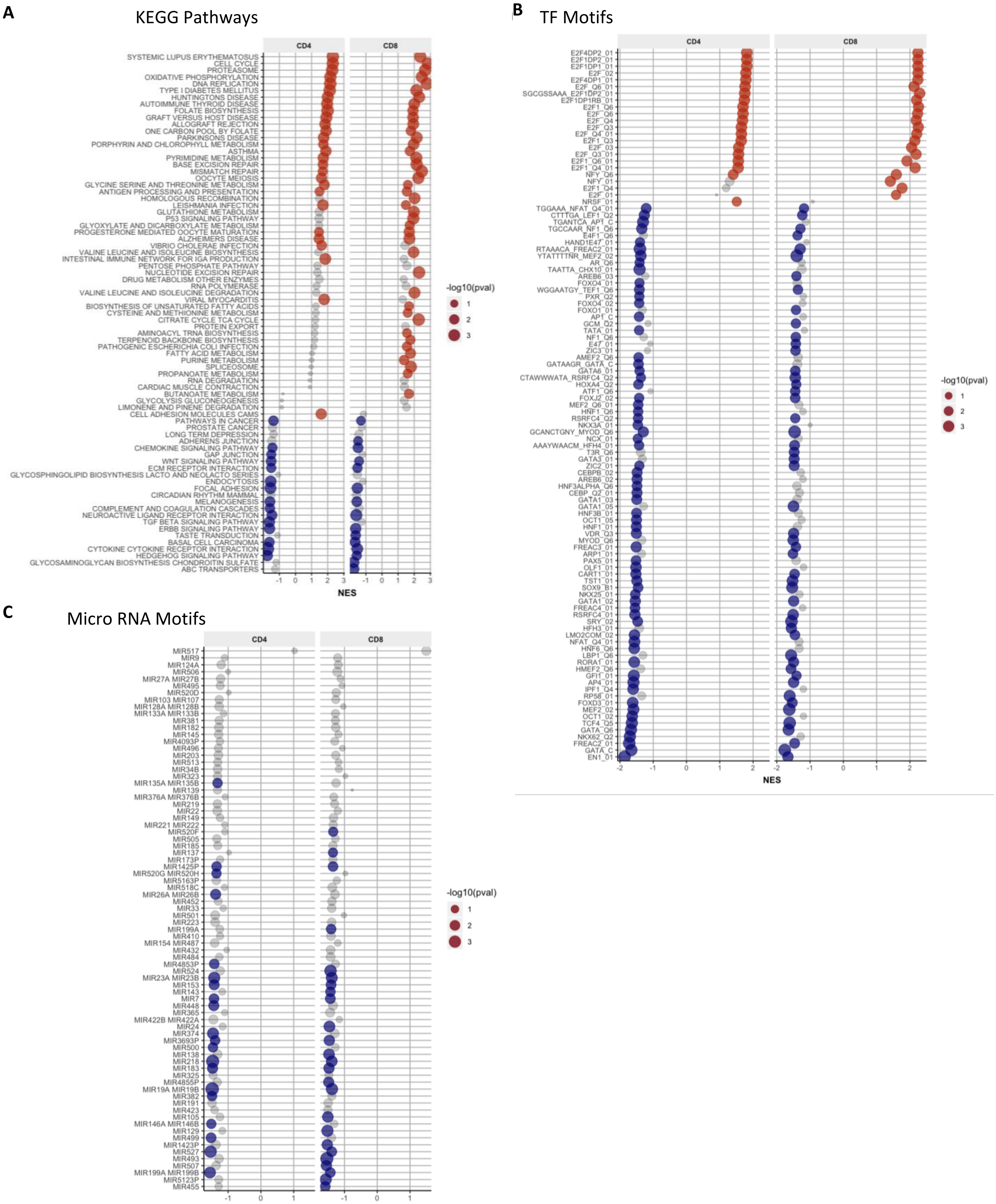

Figure S6. Gene Set Enrichment Analysis, Related to figure 7 (A) GSEA results of selected MSigDB KEGG pathway gene sets. GSEA of selected MSigDB Transcription factor binding motif gene sets **(B)** and selected MicroRNA binding motif sets **(C).** These motif sets comprise of genes containing at least one motif for the binding site of the transcription factor or MicroRNA**.** Shown are selected sets having FDR <0.2 in either CD4 or CD8 D12 vs HD comparisons. Colored circles indicate significant enrichments (FDR<0.1) towards genes upregulated (Red) or downregulated (blue) at D12 compared to HD.

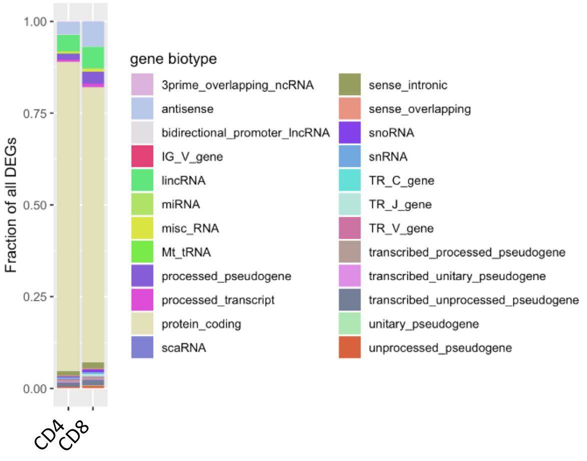

Figure S7. Proportions of gene biotypes in day 12 post autograft vs healthy donor differentially expressed genes, Related to figure 7. Proportions of annotated gene biotypes evident within genes identified as being differentially expressed in CD4 and CD8 effector T cells.

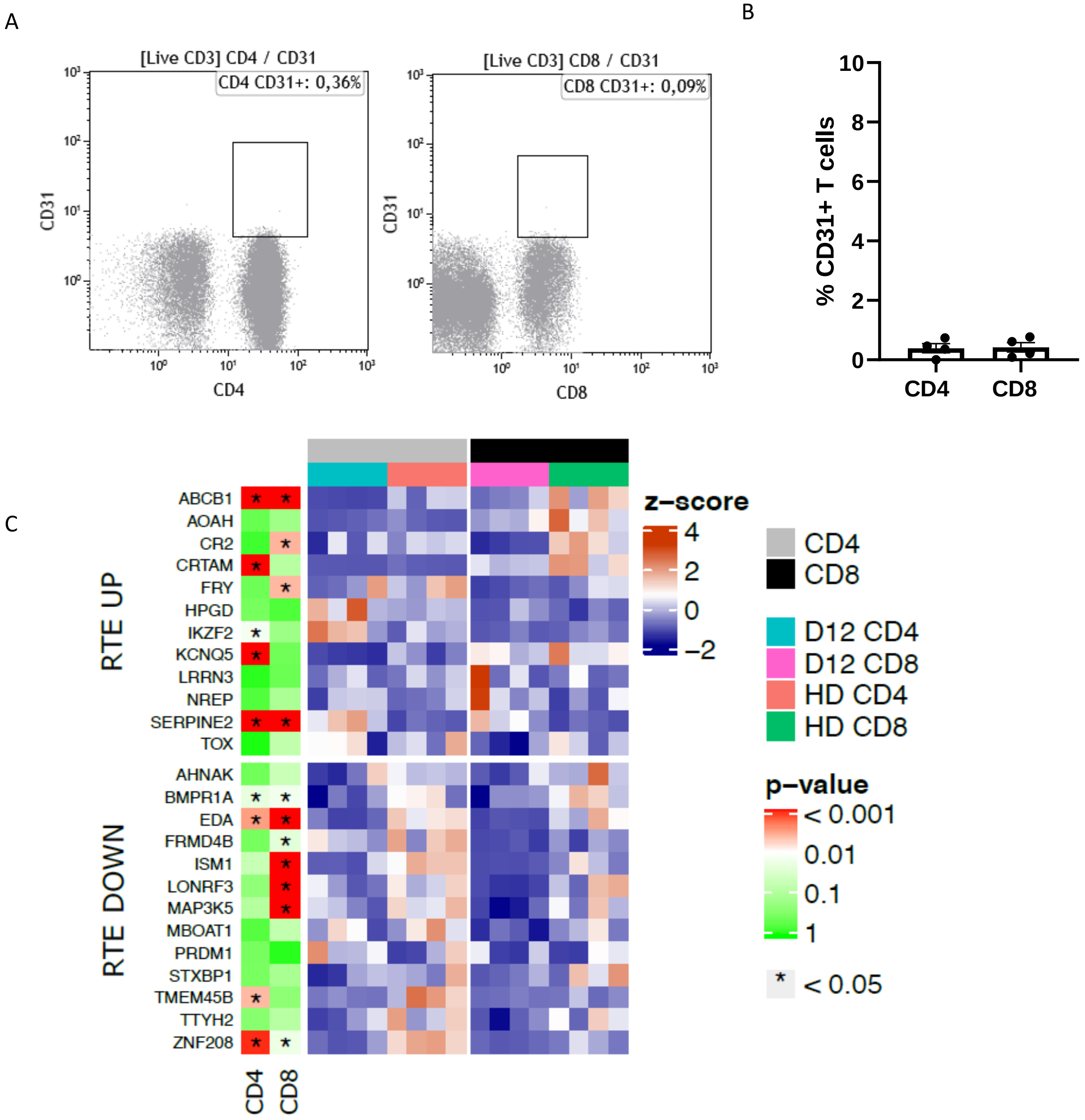

Figure S8. No phenotypic or transcriptional evidence to support presence of recent thymic emigrants is present at day 12 post-transplant, Related to figure 7 (A) Representative FACS plots depicting CD31 staining on CD4+ T cells and CD8+ T cells. (B) Percentage of CD31+ CD4 and CD8 T cells at day 12 post-transplant. (C) Expression profile of recent thymic emigrant (RTE) signature genes (Pekalski et al., 2017) in CD4/CD8 effector T cells from D12 post-autograft patients and healthy donors. p-values are from D12 vs HD differential expression analysis and are corrected for multiple testing.

Table S1. **Patient characteristics, Related to figure 1-7** (A) Characteristics of all patients included in the study (B) Characteristics of patients included in the transcriptomics analysis

		A	B
**Factor**	Characteristic	N (%)	N(%)
**Gender**	Male	89 (64%)	2(50%)
	Female	51 (36%)	2(50%)
**Median age (range)**		62 (17-74)	60(53-70)
**Diagnosis**	Myeloma	98 (70%)	4(100%)
	Lymphoma	40 (28%)	0(0%)
	Other	2(1%)	0(0%)
**Conditioning**	Melphalan	101 (72%)	4(100%)
	LEAM	14 (10%)	0(0%)
	BEAM	22 (15%)	0(0%)
	Cyclo/TBI	1 (1%)	0(0%)
	BCNU/Thiotepa	3 (2%)	0(0%)
**CMV status**	Positive	69 (49%)	0(0%)
	Negative	70 (50%)	4(100%)
	Equivocal	1 (1%)	0(0%)

BEAM: carmustine (BiCNU), etoposide, cytarabine (Ara-C, cytosine arabinoside), melphalan; LEAM: lomustine, etoposide, cytarabine (Ara-C, cytosine arabinoside), melphalan; Cyclo/TBI: cyclophosphamide, total-body irradiation; CMV: cytomegalovirus

Transparent Methods

Sample cohort

Patients undergoing autologous stem cell transplant for myeloma or lymphoma at the Queen Elizabeth Hospital, Birmingham, UK were enrolled on the study following full written informed consent (15/WM/0194). All patient details are shown in Supplemental Table 1A. A control cohort of age-matched healthy donor samples was studied for comparison using leukocyte-enriched blood cones, residual from NHSBT donation.

Sample collection

Blood samples were collected post-conditioning but prior to stem cell infusion on day 0 (D0), and post-transplant on day 7 (D7) and on day 12 (D12). Blood was taken into sodium heparin tubes for flow cytometric analysis and serum tubes for assessment of cytokine concentrations. For all subsequent experiments, fresh blood-derived T cells were analysed. Serum was frozen and stored at –80°C for later use.

Phenotypic analysis of T cells

Peripheral blood mononuclear cells (PBMCs) were isolated by density gradient centrifugation. Mononuclear cells were surface stained with antibodies on ice for 20 minutes. T cell subsets were identified using the following monoclonal antibodies: αβTCR-Pacific blue (clone IP26, Biolegend, San Diego, US), CD4-APC/Cy7 (clone RPA-T4, BD Biosciences, New Jersey, US) and CD8 PerCPVio700 (clone BW135/80, Miltenyi Biotec, Bergisch Gladbach, Germany). Naive and memory populations were defined with CD45RA-AF700 (clone HI100, Biolegend) and CCR7-FITC (clone 150503, R&D Systems, Minneapolis, US) expression. CD25-PE/Cy7 (clone M-A251, BD Biosciences), CD127-BV510 (clone HIL-7R-M21, BD Biosciences), and CD95-AF647 (clone DX2, Biolegend) were included for phenotypic expression analysis. CD14-ECD (clone RMO52), CD19-ECD (clone

J3-119, Miltenyi Biotec) and CD56-PECF594 (clone B159, BD Biosciences) were added to gate out monocytes, B cells and NK cells. Propidium iodide (PI) was added immediately prior to acquisition to exclude dead cells. To identify Tregs, PBMCs were stained with fixable red LIVE/DEAD dye (Invitrogen, California, US) and above mentioned surface antibodies for 30 mins on ice. Cells were fixed by adding 1ml of 1X FOXP3 Fix/Perm solution (Biolegend) to each tube, vortexed and incubated at room temperature in the dark for 20 minutes, spun down and supernatant was discarded. Cells were then re-suspended in 1ml 1X BioLegend's FOXP3 Perm buffer, incubated at room temperature in the dark for 15 minutes, spun down and then re-suspended in 100 ul of 1X BioLegend's FOXP3 Perm buffer. anti-FOXP3-AF647 (clone 206D, Biolegend) was added and cells were incubated at room temperature in the dark for 30 minutes. Ki67 staining was performed at the same time along with anti-FOXP3 staining using anti-Ki67-PECy7 (clone Ki-67, Biolegend). Finally, cells were washed in PBS, spun at 2000rpm and re-suspended in 200ul of PBS. Data were acquired on Gallios followed by analysis using Kaluza software (both Beckman Coulter, Pasadena, US). The lymphocyte population was identified by forward scatter/side scatter dot plots and live T cells were identified by gating on PI– αβTCR+ cells. Clinical counts (lymphocytes per ml whole blood) were provided by the Queen Elizabeth Hospital for each patient. The absolute number of TCRαβ T cells was calculated using the clinical counts together with flow cytometric analysis of lymphoid subsets.

Quantification of serum analytes

ELISA assays (R&D systems) were performed to determine the concentration of IL-7, IL-15, IFN-γ and TGF-β in serum from patients at day 0 and day 12 or age-matched healthy donors. Plates were read using a iMark microplate reader (Bio-Rad Laboratories, California, US).

RNA sequencing, differential expression and functional enrichment analysis

RNA was isolated from FACS-sorted CD4+ and CD8+ effector (CD45RA-CCR7-) T cells isolated from healthy donor PBMCs (n=4) and day 12 post-autograft PBMCs (n=4). Patient characteristics are described in Table S1-B.

Total RNA isolation was performed following the manufacturer's instructions using the RNeasyPlus Micro Kit (Qiagen, Cat. No. 74134). In brief, the sorted cells were re-suspended in 350 µL Buffer BLT and vortexed. Equal amount of 70% Ethanol was added and the suspension was transferred to Rneasy spin column and spun for 15s at 10,000rpm. The flow through was discarded and the column was washed with Buffer RW1 and Buffer RPE. The final RNA was eluted in 30 µL RNase-free water. 10ng of RNA was used to prepare RNA libraries were prepared using a TruSeq Stranded Total RNA Library preparation kit and paired end sequencing performed on Illumina NextSeq. Reads were aligned to the human reference genome (hg19) with STAR aligner (Dobin, Davis et al. 2013) and raw read counts quantified with HTSeq (Anders, Pyl et al. 2015). Normalisation and differential expression analysis was performed using the DESeq2 package (Team 2013). Genes were considered differentially expressed if the adjusted p value (Benjamini Hochberg procedure (Benjamini and Hochberg 1995) was < 0.05 and absolute log2FC > 1.5. Gene Set Enrichment analysis was performed on published gene sets from MSigDB (Liberzon, Subramanian et al. 2011) using the R package FGSEA (Sergushichev 2016). Pathway analysis was performed on lists of differentially expressed genes using gProfiler (Raudvere, Kolberg et al. 2019) Visualisations were generated using the R package ggplot2 (Wickham 2016).
